# Observation of the Mating Behavior of Honey Bee (*Apis mellifera* L.) Queens Using Radio-Frequency Identification (RFID): Factors Influencing the Duration and Frequency of Nuptial Flights

**DOI:** 10.3390/insects5030513

**Published:** 2014-07-01

**Authors:** Ina Monika Margret Heidinger, Marina Doris Meixner, Stefan Berg, Ralph Büchler

**Affiliations:** 1 LLH, Bee Institute, Erlenstrasse 9, D-35274 Kirchhain, Germany; E-Mails: marina.meixner@llh.hessen.de (M.D.M.); ralph.buechler@llh.hessen.de (R.B.); 2 Bavarian State Institute for Viticulture and Horticulture, Bee Research Center, An der Steige 15, D-97206 Veitshöchheim, Germany; E-Mail: stefan.berg@lwg.bayern.de

**Keywords:** *Apis mellifera*, honey bee queen, mating behavior, nuptial flight, radio-frequency identification (RFID)

## Abstract

We used radio-frequency identification (RFID) to record the duration and frequency of nuptial flights of honey bee queens (*Apis mellifera carnica*) at two mainland mating apiaries. We investigated the effect of a number of factors on flight duration and frequency: mating apiary, number of drone colonies, queen’s age and temperature. We found significant differences between the two locations concerning the number of flights on the first three days. We also observed an effect of the ambient temperature, with queens flying less often but longer at high temperatures compared to lower temperatures. Increasing the number of drone colonies from 33 to 80 colonies had no effect on the duration or on the frequency of nuptial flights. Since our results agree well with the results of previous studies, we suggest RFID as an appropriate tool to investigate the mating behavior of honey bee queens.

## 1. Introduction

Honey bee (genus *Apis/Apis mellifera*) queens are polyandrous and mate only at one period in their life [1,2,3,4,5]. Usually within 1 week after emergence [6,7], mating takes place in free flight, supposedly at mating leks that are also known as drone congregation areas (DCA) [6,8,9]. At these sites that have been shown to be temporally stable [10,11], between 8000 to 15,000 drones can be present during specific times of the day [12,13]. Usually, queens perform a small number short flights of a few minutes for orientation, prior to true nuptial flights which are usually longer [14,15,16]. A queen can mate several times during a single flight [17,18,19], but queens often perform several consecutive nuptial flights on the same day or on different days [14,17,20,21,22]. Between one and six flights on a couple of days have been reported [14,21,23,24,25]. The number of drones a queen mates with ranges between 6 and 26, with an average of 12–14 [26,27,28]. The number of spermatozoa in the spermatheca [20,29] and the number of matings itself [17] have been hypothesized to serve as a signal for undertaking an additional flight or to start egg laying. In contrast, Tarpy and Page [30] state that queens have no control over the number of times they mate and that the high mating frequencies of honey bees is simply a stochastic by-product of mating behavior and mate availability. Several factors have been identified to influence the mating behavior of honey bee queens, most prominently among them the age of the queen [16], but also environmental conditions such as temperature, wind, and cloud cover [23,31,32,33]. The number of available drones within the flight range of a queen has also been discussed as an important variable to influence number and duration of individual nuptial flights [14]. Since mating is fatal to the males which leave part of their endophallus in the sting chamber of the queen, the mating success of a flight can often be determined upon return of a queen to her colony [34,35].

The mating success and the number of mating partners of a given queen can be assessed via indirect methods such as counting spermatozoa in the spermatheca [14,18,20] or determining the number of patrilines by microsatellite analysis [26,28,36]. In contrast, the mating behavior itself, taking place in free flight, remains hardly accessible to observation, despite the development and availability of advanced methods and techniques for its study. While early reports were based upon direct observation of free flying or tethered queens [37,38,39,40], subsequent studies used wooden queen dummies or pheromone-treated preserved queens fixed to a rotating beam on a mast to stimulate and study the mating behavior of flying drones [34,41]. The flight activity and congregation behavior of drones has also extensively been studied using pheromone traps [42] attached to helium filled balloons [13,43,44] and by X-band radar [45,46,47]. While sexually mature drones fly frequently and continuously until they mate [48], an individual queen performs comparatively few flights [23,24]. The study of queens’ behavior requires continuous observation of the flight entrance of the respective mating nuc(s) and/or the use of a queen excluder to prevent an unobserved departure. Although technical means to monitor the departure and arrival of queens such as photoelectronic control were developed comparatively early [49], the standard method still remains direct observation of the nuc entrance combined with a queen excluder. This method limits the number of nucs that can be observed simultaneously and it may also interfere with the behavior of the queen [23,33], since she has to wait until the observer removes the excluder, both at departure and return. 

Recently, radio-frequency identification (RFID) has been successfully used in Hymenoptera to investigate social interactions in ants [50], nest-drifting behavior in wasps [51], individual activity of bumblebees [52], and the effect of insecticides on the foraging behavior of *A. mellifera* workers [53,54]. To evaluate the suitability of this technique for the study of mating flight behavior of honey bee queens, we labeled queens (*Apis mellifera carnica*) with RFID transponders to record their departure from and return to their mating nucs that were equipped with reader modules. In this paper, we report results on duration and frequency of queen nuptial flights from RFID data. We analyze the flight behavior of our experimental queens in relation to their age and to environmental variables such as location of the mating apiary and ambient temperature. In addition, we manipulated the number of available drones in the vicinity of the mating apiaries to study the effects on queen flight duration and frequency. 

## 2. Experimental Section

### 2.1. Field Work

**Figure 1:**
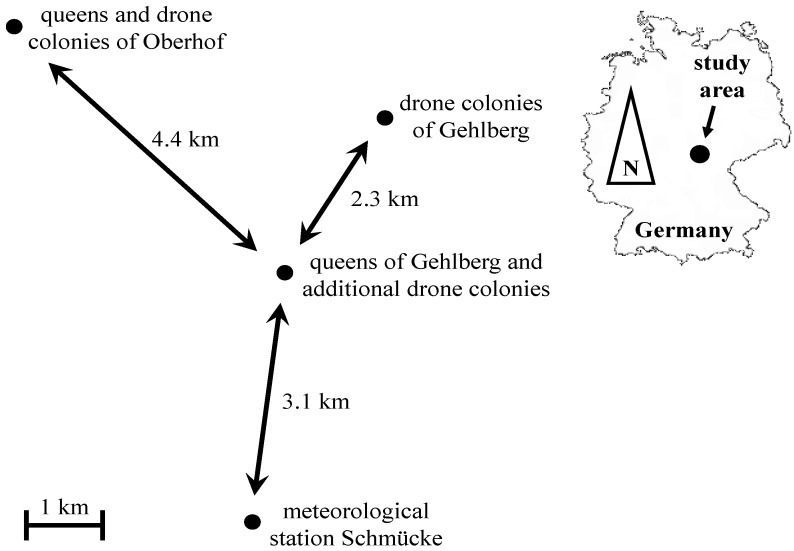
Location of the mating apiaries Gehlberg and Oberhof in the rural district ‘Ilm-Kreis’ in Middle-Thuringia, Germany.

The fieldwork was conducted at two mating apiaries situated in the rural district ‘Ilm-Kreis’ in Middle-Thuringia, Germany (Figure 1). One apiary is located near Gehlberg (50°40'57'' N, 10°46'30'' E, altitude 605 m) and the second one is located near Oberhof (50°42'36'' N, 10°43'50'' E, altitude 798 m). The distance between the two mating apiaries is 4.4 km. Each mating apiary is protected by an area of 154 km^2^ (a radius of 7 km around each station) where according to regional law beekeepers are allowed to keep only colonies headed by queens provided by the respective mating apiary. During the whole experiment, 13 drone colonies (Gehlberg colonies) were situated 2.3 km to the north-east of the nucs with virgin queens at Gehlberg and 20 drone colonies (Oberhof colonies) were located close to the nucs with virgin queens at Oberhof (Figure 1). In calendar weeks 24 and 27 of 2011 (Table 1), we placed two trailers with 47 additional drone colonies next to the queens at mating apiary Gehlberg (about 100 m, Figure 1). These additional colonies were removed again before new groups of queens were placed at the mating apiaries.

**Table 1:** Number of drone colonies at mating apiaries Gehlberg and Oberhof during the experiment.

Calendar week	Gehlberg	Oberhof
22	13	20
24	13 + 47	20
26	13	20
27	13 + 47	20

In each experiment week (Table 1), we placed eight nucs with virgin sister queens at each mating apiary. These queens were individually marked with radio-frequency identification (RFID) transponders (mic3-TAG 64RO, carrier frequency: 13.56 MHz, Microsensys GmbH, Erfurt, Germany). The dimensions of the tags were 1 × 1.6 × 0.5 mm with a weight of 2.4 mg. A reader module (iID MAJA reader module 4.1, Microsensys GmbH, Erfurt, Germany) connected with a host (iID HOST type MAJA 4.1, Microsensys GmbH, Erfurt, Germany) was attached to the entrance of each mating nuc. Every time a tagged queen was leaving or entering the mating nuc, the RFID tag of the queen was registered by the reader module and the date, time, reader- and tag ID-number were stored on the host. The data were downloaded from the host and processed manually using Microsoft Excel 2010. Climate data from the meteorological station Schmücke were used to determine the impact of temperature on the nuptial flight duration and flight frequency.

### 2.2. Data Analysis

The number and duration of the flights of each queen were calculated from the RFID readings. We excluded flights of less than three minutes or more than 60 min from the analysis. We counted flights of less than three minutes as orientation flights rather than as nuptial flights [14]. Nuptial flights of more than one hour duration also appear unlikely [23]. Occasionally, queens do not enter their mating nuc immediately after returning, but instead cluster together with worker bees in front of the entrance (own observation). In the four weeks of the experiment, it was not always possible to provide queens of the same age. Therefore, and to avoid small sample sizes, queens were pooled into three categories in regard to their age when they left their nucs for a flight (Table 2). Likewise, since not all queens flew an equal number of times, the ranks of flights were also pooled into four categories (Table 2). The groups of queens in different experimental weeks differed in the number of days they were able to fly (due to environmental reasons such as weather, or due to reasons connected to experiment logistics). For this reason, for the analysis of flight frequency only the flights of the first three days of all queens were used. The mean temperature for these first three flying days was calculated from the hourly measurements between 12:00 and 17:00.

**Table 2:** Grouping of the data concerning the queen’s age and the sequence of flights. All queens were kept in a dark room at 15 °C until they were brought to the mating stations.

Factor	Category 1	Category 2	Category 3	Category 4
Age of the queens	5 to 9 days	10 to 13 days	14 to 17 days	---
Sequence of flights	1st flights	2nd flights	3rd flights	4th to 7th flights

We analyzed our data using univariate, general linear models (GLM). For the comparison of two or more samples, we used Mann-Whitney-tests and Kruskal-Wallis-tests (since the data were not normally distributed), respectively. P values of multiple pairwise tests were obtained after a sequential Bonferroni adjustment [55]. We performed all analyses using the statistics software package SPSS 19.0.0 (IBM).

## 3. Results and Discussion

### 3.1. Survival Rates and General Flight Behavior of the Queens

**Figure 2:**
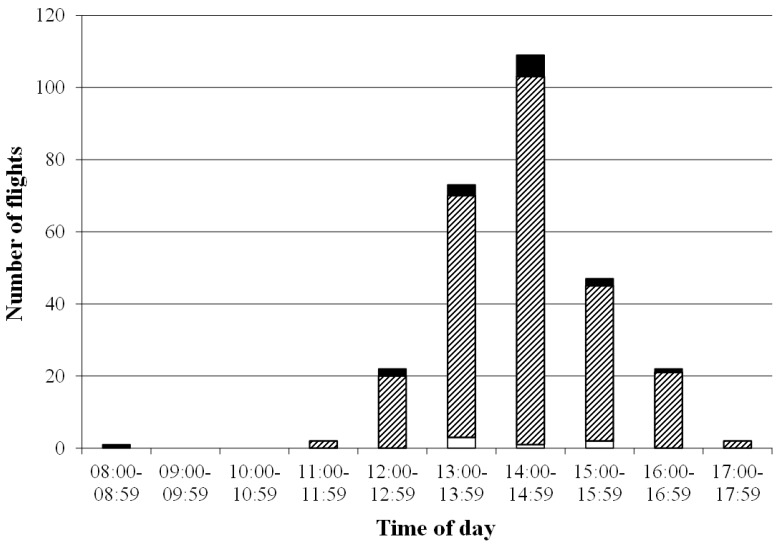
Distribution of departures over time of day. Flights with duration less than three and more than 60 min are indicated in white and black bars, respectively. Flights from 3 up to 60 min are shown in hatched pattern.

**Figure 3:**
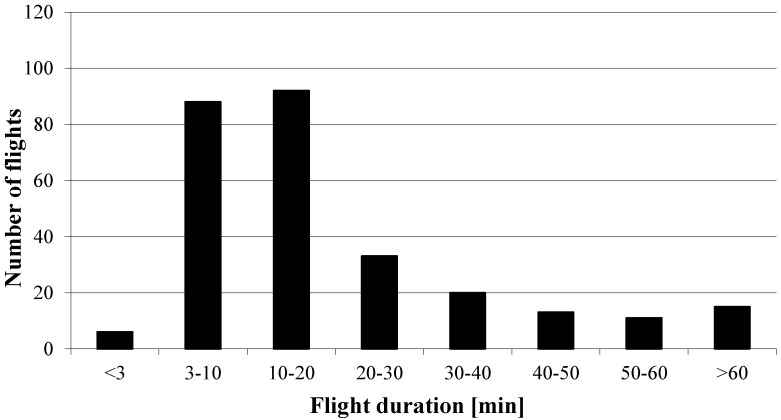
Number of flights with a specific duration.

From the total of 64 queens observed, 11 (17.19%) did not return to their mating nucs (Table 3). Three of the lost queens tried to enter a foreign mating nuc and one of them was recorded in the wrong entrance at two consecutive days. Two queens (3.13%) were not recorded at all during the whole experiment; however, they started egg laying afterwards. On average, the queens left their nucs on 2.20 ± 0.98 flying days (min. 1; max. 5), with most of the nuptial flights (82.49%) taking place between 13:00 and 16:00 h (Figure 2). The earliest and latest departure was recorded at 11:50 and 17:38 h, respectively. The average total number of recorded flights per queen was 5.04 ± 3.11 (min.1; max. 16), with a maximum of seven flights of one queen on one day. The mean daily number of nuptial flights per queen was 2.30 ± 1.35, with a mean duration of 17.69 ± 13.19 min (min. 3.08; max. 57.07; Figure 3).

**Table 3:** Number of queens with recorded nuptial flights, number of mated (queens which started egg laying) and lost queens (queens which did not return from the mating station) for each location and experiment week. Two of the mated queens were not recorded at all.

Calendar week	Total number of monitored queens	Queens with recorded flights	Mated queens	Lost queens
Gehlberg				
22	8	7	7	1
24	8	7	7	1
26	8	5	5	3
27	8	3	3	5
Oberhof				
22	8	7	7	1
24	8	6	8	0
26	8	8	8	0
27	8	8	8	0

These results are in accordance with previous studies. Queen losses of 10% to 20% during nuptial flights due to weather conditions, predation or difficulties in returning to their mating nuc are not unusual [17,56]. In general, honey bee queens perform several flights on a couple of days. Schlüns *et al*. [17] and Woyke [20] reported up to three flights for queens on the mainland. Alber *et al*. [23] observed one to four mating flights (queens returned with a mating sign) on one to three days for queens on an island. Verbeek [33] reported up to 10 flights on three days and a mean number of flights per queen of 12.5 (mainland) which appears to be rare for queens on mainland mating apiaries. However, this many flights and even more have been reported for queens on islands [23,33]. Our results are well in agreement with previously reported observations from various climates and at various latitudes (northern hemisphere) in that *A. mellifera* queens and drones take nuptial flights between 12:00 and 17:00 h with a maximum flight activity between 13:00 and 16:00 h [23,24,31,33,48,57,58,59,60]. Data on the flight duration of honey bee queens provided by different authors vary considerably between 2 and 57 min [21,33,61,62] with a mean duration between 7 and 26 min [1,14,18,33]. 

### 3.2. Duration and Frequency of Queen Nuptial Flights

**Figure 4:**
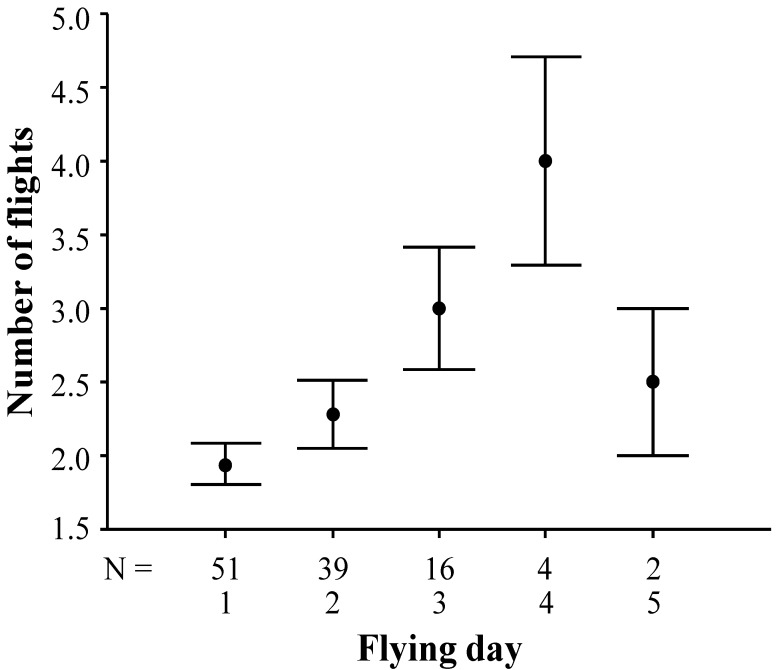
Comparison of the daily number of nuptial flights between different consecutive flying days. Arithmetic mean and standard error are given for each day. N is the number of queens flying on one to five consecutive days.

First, we considered all flying days of all queens, and observed no significant differences concerning the number of flying days per queen (Mann-Whitney-test; U = 264.50; *p* = 0.270) as well as the total number of nuptial flights per queen (Mann-Whitney-test; U = 229.00; *p* = 0.085) between the two mating apiaries. When queens of both mating apiaries were considered together, the number of flights per day differed significantly between different consecutive flying days (Kruskal-Wallis-test; Chi^2^ = 12.30, *p* = 0.015, df = 4), with the number of flights per day increasing until day 4 and then decreasing again. Significant differences were observed between days 1 and 3 (Mann-Whitney-test; U = 250.00; *p* = 0.015), days 1 and 4 (Mann-Whitney-test; U = 19.50; *p* = 0.005), and days 2 and 4 (Mann-Whitney-test; U = 25.50; *p* = 0.023; Figure 4). However, after sequential Bonferroni adjustment of the *p*-values for multiple testing, only the difference between the number of nuptial flights on days 1 and 4 remains significant.

**Figure 5:**
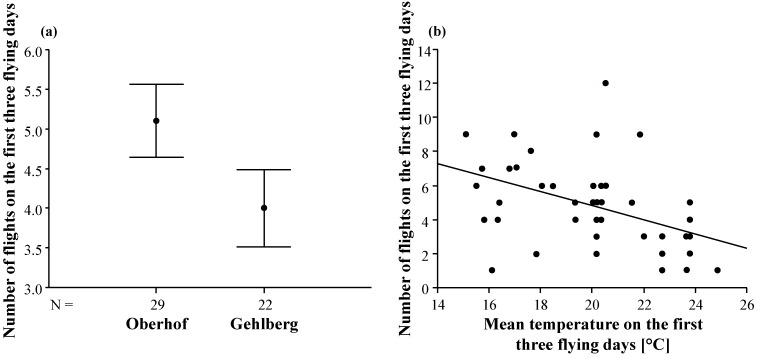
(**a**) Comparison of the total number of nuptial flights (on the first three flying days) at Oberhof and Gehlberg. Arithmetic mean and standard error are given for each mating apiary. N is the number of queens flying at Oberhof and Gehlberg respectively. (**b**) The total number of nuptial flights of each queen in relation to the mean temperature for the first three flying days.

Then, we restricted the further analysis of flight frequency to the first three flying days, since groups of queens differed in the number of days that flights were possible (see Section 2.2). The number of nuptial flights on the first three flying days differed significantly between the two locations, with queens of Oberhof flying more often than queens of Gehlberg (Table 4, Figure 5a). In addition, the total number of flights of the queens was higher on days with lower mean temperatures than at higher temperatures (Table 4, Figure 5b). However, we observed no effect of the number of drone colonies on the total number of nuptial flights of a queen (Table 4).

**Table 4:** Effects of mating apiary, mean temperature (included as a covariate) and number of drone colonies on the total number of nuptial flights of the queens on the first three flying days. Type III sum of squares, denominator degrees of freedom (Den. d.f.), mean square, *F*-value and *p*-value are given for each factor.

Source	Type III sum of squares	Den. d.f.	Mean square	*F*-value	*p*-value
Model	1197.26	5	239.45	57.75	0.000
Mating apiary	21.90	1	21.90	5.28	0.026
Mean temperature	19.89	1	19.89	4.80	0.034
Number of drone colonies	3.67	1	3.67	0.88	0.352
Mating apiary x Number of drone colonies	9.17	1	9.17	2.21	0.144
Error	190.74	46	4.15		
Total	1388.00	51			
R^2^ = 0.863; R^2^ corrected = 0.848					

**Figure 6:**
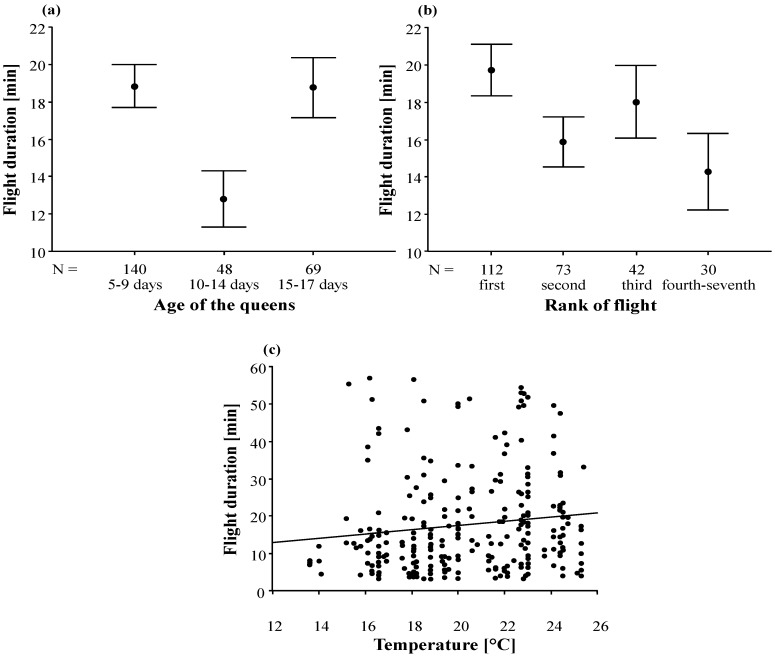
(**a**) Duration of the nuptial flights depending on the age of the queen. Arithmetic mean and standard error are given for each age class. N is the number of flights in the dataset. (**b**) Comparison of the duration of the first, second and third nuptial flights on a given day and all other flights of the same day. Arithmetic mean and standard error are given for each class. N is the number of flights in the dataset. (**c**) The nuptial flight duration in relation to the temperature when queens left their nuc.

In contrast to the number of nuptial flights of all queens, their mean duration did not differ significantly between the two mating apiaries (Table 5). The mean daily nuptial flight duration of each queen did not differ between flights taken on different days (Kruskal-Wallis-test; Chi^2^ = 2.58, *p* = 0.631). We also found no effect of the number of drone colonies on the duration of the nuptial flights (Table 5). However, flight duration significantly depends on the age of the queen (Table 5, Figure 6a), with the youngest (<9 days) and oldest (>15 days) queens flying significantly longer than queens of medium age (10 to 14 days). Flight duration also depends on the rank of the flight (Table 5, Figure 6b), where the first flights were the longest ones. In addition, the duration of the nuptial flights significantly increased with increasing temperature (at departure; Table 5, Figure 6c).

**Table 5:** Effects of queen’s age, mating apiary, number of drone colonies, rank of consecutive flights and temperature (at departure; included as a covariate) on the duration of the nuptial flights. Type III sum of squares, denominator degrees of freedom (Den. d.f.), mean square, *F*-value and *p*-value are given for each factor.

Source	Type III sum of squares	Den. d.f.	Mean square	*F*-value	*p*-value
Model	83,767.52	10	8376.75	50.24	0.000
Age of the queens	1198.90	2	599.45	3.60	0.029
Mating apiary	34.24	1	34.24	0.21	0.651
Number of drone colonies	116.40	1	116.40	0.70	0.404
Rank of flight	1359.03	3	453.01	2.72	0.045
Temperature	650.91	1	650.91	3.90	0.049
Mating apiary x Number of drone colonies	47.45	1	47.45	0.29	0.594
Error	41,187.13	247	166.75		
Total	124,954.65	257			
R^2^ = 0.670; R^2^ corrected = 0.657					

Several studies identified environmental conditions such as temperature, wind, and cloud cover as important factors influencing the mating behavior of honey bees [31,32,33,60,63]. Alber *et al*. [23] showed that matings hardly ever occur at temperatures below 20 °C with overcast sky and wind velocity above 30 km/h. According to Lensky and Demter [31] high wind velocities (9–14 km/h) in combination with low temperatures (15 °C–20 °C) lead to an increase of short queen flights. In our study, the flight duration also decreased with decreasing temperatures whereas the flight frequency increased. The mating behavior of honey bee queens can also vary between different types of mating apiaries. For example, nuptial flights of queens on island mating apiaries are shorter and more frequent than on mainland mating apiaries [33,64]. In addition, queens on island mating apiaries mate less often compared to queens on the mainland [28]. These differences have been considered to be caused by different climatic conditions (especially wind velocity) between islands and the mainland [28,33,64]. Our study was conducted at two mating apiaries separated by a distance of 4.4 km only. Nevertheless, local climate conditions are likely to differ between the two locations, especially since the altitude of Oberhof is about 200 m higher than Gehlberg. This difference may explain the different flight frequencies at the two apiaries, with queens of Oberhof flying more often than queens of Gehlberg.

The number of available drones in the environment has been hypothesized to influence the mating flights of honey bee queens, although the reports of different author teams differ in their conclusions. While Koeniger and Koeniger [14,57] observed an increase in mean flight duration from 13.7 ± 6.1 min with plenty of drones (>10,000) to 21.8 ± 9.67 min with few (~2500) drones, Woyke [18] showed the opposite effect, with an abundance of drones in close proximity to the queens leading to longer nuptial flights. However, the nuptial flights of the queens from an apiary with plenty of drones were not more effective (concerning the amount of semen queens received) than those of queens from an apiary where no colonies with drones were present within 2.5 km [18]. Neumann *et al.* [28] compared queen mating frequencies and found no effect with numbers of drone colonies varying between 10 and 42. In our study we increased the number of drone colonies from 33 to 80 colonies and found no effect, neither on the flight duration nor on the flight frequency. Possibly, 33 drone colonies are already enough for an excess supply of drones so that the effect of additional drone colonies is rather weak. 

We found no previous studies reporting differences in flight duration in queens of different ages, where we observed a significant effect. The flights of both the youngest and oldest queens were significantly longer than those of medium-aged queens. However, due to reasons connected to experiment logistics, our groups of queens in different experimental weeks were not always all of the same age. Therefore we cannot exclude the possibility that the effect of the queen’s age may have resulted from differences between different experimental weeks. To confirm our observation it will thus be necessary to observe queens of different age simultaneously at the same mating apiary.

Direct visual observation of queens is time-consuming and the number of individuals which can be monitored simultaneously, as well as the time span during which this can be done, is limited. To prevent unobserved nuptial flights, queen excluders are used for direct observations which have to be checked continuously by a patrolling observer. Both the presence of an observer and the queen excluder itself, can disturb the queens and alter their behavior [23,33]. In contrast, use of the RFID technique provides a suitable method to monitor several individuals simultaneously without disturbing them. Of course, the use of RFID does not permit to detect whether a queen returns with a mating sign, so it cannot be determined whether she mated on a flight or not. However, even with direct observation one cannot always be certain whether a queen was successful on her nuptial flight, as mated queens sometimes (8% to 36%) return without a mating sign [1,23,65]. One can only be sure that a queen returning with a mating sign mated with at least one drone. A more serious problem occurring with RFID is the fact that sometimes a queen can pass the reader module without being registered. Three of our queens were not recorded at all during the whole experiment; however, they started egg laying afterwards. Although it is possible that they lost their transponder, we also found that the orientation of the transponder relative to the reader modules influences the readings. It may thus be possible that queens leave unregistered when they pass the entrance in a position that is unfavorable for a reading. Another technical problem was that we sometimes could not unambiguously decide whether a queen left her mating nuc for a nuptial flight or if, after having passed the reader module, she did not leave after all but returned into the nuc instead. Both these difficulties can be solved by use of reader modules with two antennae. Queens passing such a reader module are registered twice, which enables the observer to determine the direction of the movement [52,66].

## 4. Conclusions

Radio-frequency identification (RFID) is an appropriate tool to investigate the duration and frequency of nuptial flights of honey bee queens, since our results agree well with the results of previous studies. Marking the queens with RFID transponders does not lead to increased queen losses. In our study, the number of queens which did not return to their mating nucs was within the range of losses reported by other authors, and rather seems to depend on the mating apiary (10 queens were lost at Gehlberg and 1 queens at Oberhof). To reduce the rate of ambiguous readings, reader modules with two antennae should be used.
